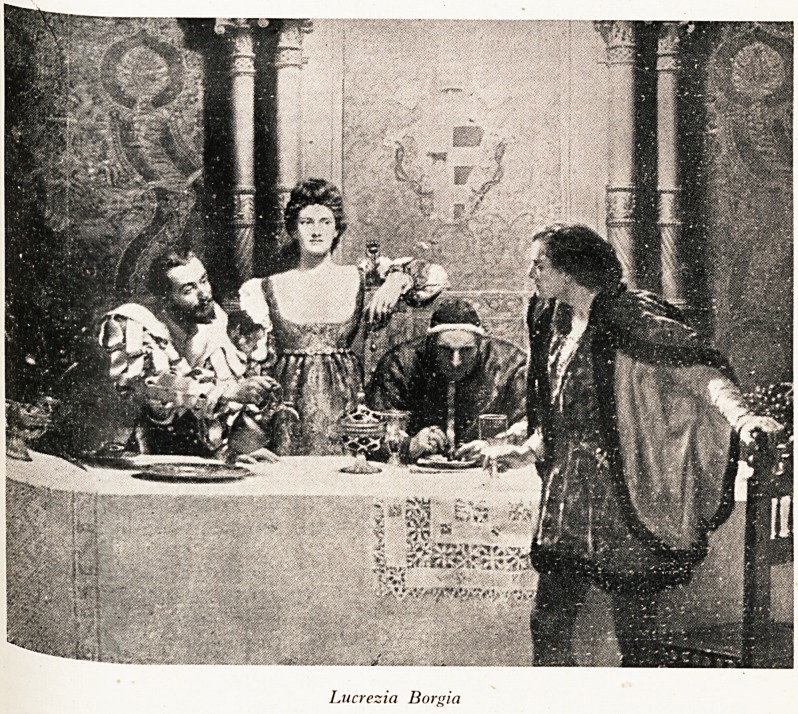# Damned Drugs (Part I)

**Published:** 1954-07

**Authors:** G. E. F. Sutton

**Affiliations:** Consultant Physician, Bristol Royal Hospital


### DAMNED DRUGS

### PART I

### The Presidential Address, given at The Royal Fort, Bristol, on 8th October, 1952 at the opening of the Seventy-Fourth Session of the Bristol Medico-Chirurgical Society

BY

DR. G. E. F. SUTTON, *M.C.*, M.D., F.R.C.P.
*Consultant Physician, Bristol Royal Hospital*

In *Cymbeline* the king's physician thus describes the love of the former queen:

> More sir, and worse, she did confess she had
> For you a mortal mineral which being took
> Should by the minute feed on life and lingering
> By inches waste you; In which time she proposed
> By watching, weeping, tendance, kissing, to
> O'ercome you with her show.

Even so throughout the long and chequered history of man's career on " this darkling plain swept with confused alarms of struggle and flight", there have been men and women who have brewed strange brews, either to deal death swift and sudden, or to encompass their victim more slowly and even more insidiously than the silent march of some fell disease.

Poisons were well known among the ancients, and centuries before the Christian Era the Sanskrit writings show that the Hindoos were familiar with animal, vegetable and mineral poisons as well as with their antidotes. Thus it was written: " It is necessary for the practitioner to have knowledge of the different poisons and antidotes, as the enemies of the Rajah—bad women and ungrateful servants—sometimes mix poison with food. Food which is thus suspected should first be given to certain animals and if they die it is to be avoided."

The most notorious as well as the most remorseless of the Greek poisoners was Olympias, the wife of Philip of Macedon and mother of Alexander the Great. Among many others, she was responsible for the death of Aridaeus, the natural son of Philip, his wife Eurydice and Nicanor. To those who were imprisoned at her instigation she kindly allowed the choice between the sword, a rope, and a cup of hemlock.

Later, in the first century of the Christian Era, we find the loathsome Locusta using slow and wasting poisons to destroy her numerous victims. She dispatched Claudius by order of Agrippina, and, later, Britannicus by order of Nero. Indeed the latter was so impressed by her talent that he showered gifts upon her and insisted on her teaching a selected class in order that her art might be perpetuated.

In the year 331 B.C., an epidemic broke out in Rome which was at first supposed to arise from corrupt air until it was observed that the illness was confined to the principal patricians. Even so, further inquiries would not have been made were it not for the sudden and unexpected accusation by a female slave of more than twenty Roman ladies of poisoning. There followed a widespread investigation and inquiry, as a result of which no less than 366 ladies were accused of poisoning. Two of them, Cornelia and Sergea, both distinguished patricians, were caught in the act of preparing their fatal potions, but, when they were led before the popular assembly, they swore that their preparations were but harmless remedies and promptly accused the slave of being a false witness. She, however, had the wit to suggest that in proof of their statement the ladies should swallow these so-called harmless remedies themselves. This they did and were thus fortunate in avoiding a painful trial and a more shameful death.

Turning over the pages we next dip into the fifteenth century in Italy where flourished the notorious Venetian poisoners. The infamous Council of Ten here met to consider the removal of emperors, princes, powerful nobles and other high dignitaries by the subtle and time-honoured method. Records still exist of their proceedings and they show that whenever the deed was done the simple and significant word " Factum " was appended to the victim's name.

On December 15th, 1643, John of Regula, a Franciscan friar, offered the Council of Ten a selection of poisons and calmly informed them that he would remove anyone they desired for a suitable sum. His terms were clearly and succinctly stated as follows: " For the first successful case a pension of 1,500 ducats a year, to be increased on the execution of the projected task." His tariff then ran as follows: " For the Great Sultan 500 ducats. For the King of Spain 150 ducats (including expenses of the journey). For the Duke of Milan 60 ducats. For the Pope 100 ducats." An addendum to his tender ran as follows: " The farther the journey and the more eminent the man, the more it is necessary to reward the toil and hardship undertaken, and, accordingly, the heavier must be the payment."

The Presidents of the Council, Guolando Duoda and Pietro Guiarini, placed his project before the members on January 4th, 1544, and on a division it was resolved to accept this patriotic offer and to experiment first on the Emperor Maximilian.

From the sixteenth century onwards, poisoning in Italy assumed endemic, and, at times, even epidemic proportions. The most notorious exponent at this time was the woman Toffana, or Toffania, who made a fortune from dispensing poisons throughout Italy. Small glass vials were dispatched bearing the image of a saint and labelled with various attractive names such as " Aquetta di Napoli " or " The Manna of St. Nicholas of Bare " and " Aqua Toffana ". Within these vials was a fluid which was supposed to be for cosmetic purposes. It was actually a concentrated solution of arsenious acid. Toffana carried out her nefarious trade from girlhood until she was nearly seventy years of age, and during this time she was instrumental in the poisoning of nearly six hundred people. The reasons for her non-apprehension for so long a time were, first, that she would deal only with individuals after due safeguards had been built up; secondly, she frequently changed her abode and adopted many disguises, and, finally, in an age in which it was rife, she made great show with superstition and religion. The Popes Pius III and Clement XIV are said to have fallen victims to her miraculous oil which was said to ooze from the tomb of St. Nicholas. When eventually the sale of Aqua Toffana was traced to her she took refuge in a convent, from which the abbess and archbishop refused to give her up, and from there she continued to sell the aqua for a further period of twenty years. " Aqua " was a suitable designation, for it was odourless, tasteless and colourless. Eventually public indignation was roused to such a pitch that the convent was broken into by a body of soldiers and she was handed over to the authorities. She was subsequently tortured until she confessed in 1709, and then, after she had been strangled, her body was thrown into the garden of the convent which had sheltered her.

Even as the syphilitic disease had spread from the Neapolitan ports across France in the fifteenth century, so did the epidemic of poisoning spread from Italy to France in the seventeenth century, and it became such a rage that in the reign of Louis XIV a special tribunal was established, the " Chambre Ardente ", for the sole purpose of suppressing and punishing this class of offender. Of the scores of poisoners who were brought to justice by this famous court, none commanded such widespread interest as that of the distinguished aristocrat, Marie-Marguerite d'Aubray, la Marquise de Brinvilliers. We see in her a woman who had every advantage in the way of high birth

PLATE XV

*Lucrezia Borgia*

and position, great wealth, influential connections, singular beauty, fascinating manners and elegant accomplishments, yet throwing it all to the winds like rain for the mere gratification of carnal passion. Her husband, the Marquis, invited a friend, Captain St. Croix, who was an officer in his regiment, to stay with them. It was the Marquise's father, Lieut. d'Aubray, who was quickly incensed with the knowledge of his daughter's indiscretions with Captain St. Croix and he it was who obtained a " lettre de cachet " and had the Captain sent to the Bastille. Unfortunately, Captain St. Croix there learnt a lot about poisons from an Italian poisoner, Exili, and he divulged the secrets to la Marquise. This " Jezebel ", as she has been described, decided to become a finished actress in the art by visiting the hospitals, particularly the "Hôtel Dieu", day after day, in the guise of a Sister of Charity, to experiment upon helpless victims. In the course of this diabolical work she managed to produce effects so cleverly that they appeared to be merely an aggravation of the symptoms which she was ostensibly endeavouring to relieve. Her gentility, her sympathy, and her tender compassion, became a byword in the " Hôtel Dieu " and upon her seemingly saintly head untold blessings were invoked by a large number of sufferers, whom she was secretly dispatching to the dead-house. Subsequently, she poisoned her father, her sister and her two brothers because they opposed her amorous intrigue. Unfortunately for her, Captain St. Croix lost his life from the inhalation of deadly fumes in his laboratory. On examining his effects the authorities came across a small box to which was attached a note requesting that after his death it should be dispatched to the Marchioness. On opening the box it was found to contain a large collection of poisons, including corrosive sublimate, antimony and opium. When the Marquise heard of the death of her lover she at once made every endeavour to obtain the box by bribing the officers of justice, but without success. She then fled to Belgium and entered a convent at Liége from which she was subsequently decoyed by an agent disguised as a monk. She was brought back to Paris, tortured into confession and finally burnt near Notre-Dame in July, 1676.

> Oh, you do bear a poison in your mind that would
> not let you rest in paradise.

Even a brief account of poisoners in these times would be incomplete without some description of the hateful Borgias [see Plate XV]. They were of Spanish origin and came to Italy in the time of Pope Calixtus III about the year 1455. Rodrigo was the first to become notorious. He was born in 1431 and after serving for some time as a soldier, he entered the priesthood, and somehow rose to be head of the Church as Pope Alexander VI. He had five children by his mistress, Vanozza de Cattanei; of whom *Cesare*, afterwards Duke of *Valentinois*, and *Lucrezia*, (who became the Duchess of Ferrara) were notorious.

When Rodrigo became Pope Alexander VI he bestowed honours and titles on all his children, with the exception of Cesare, whom he had specially reserved for the purple. In order to do this he had first to remove the stain of his birth and he did so most effectively in a bull dated October 17th, 1480, declaring that Cesare was the son of Vanozza and Dominico d'Arignano. Later, in 1498, another youth called " Romano" was introduced to the household and the pope, declaring him to be Cesare's son, created him Duke of Nepi. The Pope himself was, however, Romano's real father, but his mother remains unknown.

Cesare's carrer of crime seems to have come to the fore on account of his jealousy over his sister Lucrezia. The latter's first marriage had been dissolved by the Pope and she then married Alfonso, Duke of Bisceglie, the natural son of Alfonso II, King of Naples. Soon after the birth of their first child the Duke was attacked by ruffians and severely wounded. His liquidation is described graphically by the chronicler as follows:

" On the night of July 15th, 1500, on which solemn ceremonies were taking place to celebrate the jubilee of the Pope, a young man staggered into the pontifical apartments endeavouring to stem with his hands a stream of blood which gushed from a large wound in his chest. It was the Duke of Bisceglie, Alfonso of Aragon, Lucrezia's second husband. Consternation was caused when it was spread abroad that a band of assassins in the pay of Cesare had attempted to assassinate him near the steps of St. Peter's when on his way to the celebration. The young man, who is said to have been of a kind and gentle nature, fell at the feet of the Pope. Lucrezia, and his sister, Sanzia, who were standing by, both fainted away and were carried into a room of the tower behind the Pope's chambers. He is said to have been nursed by the two women and to have nearly recovered when one night, in Lucrezia's absence, he was strangled with a cord in bed under the eyes of Cesare."

Some time later Cesare became violently jealous of Pedro Caldeson, who was an attendant in his sister's household. In a raging passion he pursued the man right into the pontifical apartments and assassinated him in the presence of the Pope. "Even so", says the chronicler, "that the pontifical garments were splashed with blood."

The number of deaths from poisoning attributed to the Borgias is astounding, and one has to realize that, at that time, poison had become the standard weapon in the social and political life of the country. In both political and ecclesiastic circles it procured office by removing rivals and generally smoothed the way, and made life less bothersome.

The picture painted by Apollinaire may not be quite correct, but it is accurate and lurid enough to be recorded. It is an account of a fête held in the Vineyard of St. Peter-in-Chains:

"La Vanozza receives the cardinals and the ambassadors, and, after being introduced to one another, the guests disperse about the vineyard and exchange conversation and courtesies. Later she disappears and joins Cesare in a room on the first floor of the building. She finds him with his sleeves rolled up, bent over a kneading-trough, and absorbed in his task. This room was reserved for Vanozza and Cesare only the Pope shared with them the right of entry and no one else was allowed to cross the threshold. On the floor lay several large, shallow copper dishes, some of which were entirely covered with verdigris and from which a colourless-looking liquid was being evaporated. One of these dishes was always placed nearer the fire in order that the heat might hasten the evaporation.

"As La Vanozza enters, Cesare remarks: 'Yet I forbade you to make a fire.' 'I only put a few live coals on to hasten the result', she replies, 'I did not make enough for it to be possible for the powder to scorch; if I had not done it we should not have had the powder to-day!'

"'It is not so much for fear of it scorching, but because of the cinders which mix with the powder and render it less fine', says Cesare. 'Happily, Cardinal di Riaro is short-sighted! This is quite enough for him in any case, but for the others—hand me the tart dish. It should be dry by now.'

"La Vanozza lifts the heavy, red copper dish by the two handles, and on it may be noticed a mouldiness or greenish spots caused by a settling deposit. With a hare's paw Cesare collects this powder, then, with an ivory knife, he carefully scrapes the copper and mixes the residue in a marble mortar. From it he takes in small pinches some of the powder and places it in another mortar of agate and reduces it in a pestle to an impalpable dust until it is like a morsel of polished silver.

"'Give me the manna', says Cesare. La Vanozza hands him the arsenic which he calls by this name, and he mixes some with the powder in the mortar, passing the mixture again under the pestle until thoroughly incorporated, and then, his task completed, he stands erect and exclaims: 'God said: "Let there be light", and there was light. We Borgias can say: "Let there be night", and there shall be night'. He then remarks that it is time for luncheon. La Vanozza leaves him and retraces her way. When she is gone, the copper dish being empty, he voids urine in it to replace that which has evaporated and the salts of which he had just utilized. The salts which resulted, combined with the verdigris, were then mixed with arsenic, and this formed the famous poison which the Borgias called 'La Cantarella'. That which the Borgias utilized in conjunction with arsenic without knowing it, was phosphorus, a secret which had been divulged to the Borgias by a Spanish monk who also knew the antidote for it as well as an antidote for arsenic; one sees, therefore, that they were well armed."

Alexander VI commenced a series of his horrible executions by the murder of Djem, the son of Mahomet II. The latter had been captured by the cavaliers of Rhodes and handed over to the Pope. Some little time after this, Charles VIII, King of France, demanded that Djem should be given over to him as a hostage in order that he might gain a more efficacious control over Turkey. But the wily Sultan had foreseen this move and had previously arranged a little sum of 300,000 ducats with the Pope. The result of this arrangement was that Djem died as soon as he had been handed over to the French from poison which had previously been given in his food.

As the natural heir to the cardinals, Alexander allowed them to become rich from their administrations, and when he considered that the time was ripe, "La Cantarella" brought about their speedy dispatch. It is stated in the chronicles that the cardinals of Modène, Mechiel and Arragon were thus poisoned in succession, and the Cardinal Orsini, convicted of a plot against the Pope, was duly imprisoned. A few days later he, too, died of poison and it is stated that he had been given the Borgias' famous slow poison called "Venenum Attemperatum". Finally, the cardinal's entire family were dispossessed, and the men were killed, while the women and children were poisoned.

One could go on recounting the crimes of the Borgias almost indefinitely, but you will be more interested in their respective fates. Alas, I regret to tell you that, after many vicissitudes, Cesare died in battle. As for the Pope, some chroniclers insist that he died of a fever within a day or two. Others maintain that he was a victim of his own designs. It is said that he endeavoured to dispatch the very wealthy cardinal, Datary Arian de Corneto, by poisoning some sweetmeats. The latter, however, was wily enough to offer 10,000 ducats in gold to the Pope's carver, who then agreed so to arrange the sweetmeats that the Pope himself was poisoned. According to yet another story, the Pope had invited nine cardinals to dinner hoping to dispatch them all with some poisoned wine. Unfortunately, he entered with Cesare earlier than the time fixed for the rendezvous and asked for a glass of wine for himself and Cesare. This was served by a junior servant who, knowing nothing of the plot, gave them the poisoned wine. The Pope died and Cesare recovered after a serious illness.

Both Burchard and Voltaire deny these stories and state that the weight of evidence suggests that the Pope died of a malignant fever. Whatever the cause, it seems certain that his body swelled enormously after death and that there was early severe putrefaction.

### The Case of Elizabeth Fenning

On April 11th, 1815, Elizabeth Fenning, aged twenty, was tried at the Old Bailey for having attempted to poison her employer, a Mr. Olibar Turner, his wife and his father. Elizabeth had made some dumplings for dinner and after eating them, all the family (as well as the maid herself and a young apprentice in the house) suffered from violent pains and vomiting. Mr. Turner said he suspected that they had all been poisoned with arsenic, and next morning he found white arsenic in the bowl in which the dumplings had been mixed. He then stated that a packet of arsenic labelled "Arsenick, Deadly Poison", which he left in a drawer in his office, was now missing. He also stated that he had observed that the knives they used in cutting the dumplings had turned black.

Mr. John Marshall, a surgeon who was called in, confirmed the fact that arsenic was present in the pan and that the family had been suffering from arsenical poisoning, from which they had all speedily recovered.

Despite the fact that no trace of arsenic was found in the remaining yeast and flour, the girl was found guilty and sentenced to death. The trial aroused great public interest in London, and before long there was an outburst of popular feeling against the conviction, it being generally held that the evidence was insufficient to prove the girl guilty. Several meetings of influential citizens were held agitating for a remission of the sentence. The Prince Regent, the Lord Chancellor, and the Secretary of State were duly petitioned in turn, but to no avail.

Elizabeth Fenning, at the age of twenty, was executed at Newgate on July 26th, 1815, exclaiming: "I die innocent, but God will convince you by a circumstance this day." But nothing happened on that day as Elizabeth understood it. The sun descended over the myriad mass of struggling and striving life as he had done through eons of time, *and infinitely far away there were other worlds that shone as stars in the night that was deeper than death.* But, nearly twenty years later, in 1834, her employer, Mr. Olibar Turner, died in a workhouse and confessed before his death that he had put the arsenic into the dumplings and sworn away the girl's life.

We are not told of any motive for this dastardly act, and we are left wondering whether it was the expression of a fiendish revenge for the girl having refused his amorous advances.

According to the old Hebrew word "Yom" a day is a period of time, and if the mills seemed to turn slowly, one can at least imagine something of the searing vicissitudes of life that Mr. Olibar Turner must have known to end his days in the workhouse nearly twenty years later.

I died innocent, but God will convince you by a circumstance this day.

### Catherine Wilson and Colchicum

Many are the crimes of passion and many are the crimes of greed. The case of Catherine Wilson is interesting in the latter respect.

In April, 1862, a Mrs. Connell had been separated from her husband and she invited Catherine Wilson to tea, as the latter was obviously one of those understanding women who might effect a reconciliation. Immediately after tea Mrs. Connell was taken ill. Wilson ran out, ostensibly to the nearest chemist's shop, and brought back a bottle of "Black Draught". When Mrs. Connell complained that it was very hot and burnt her mouth, Wilson said it was because she had warmed it and begged her to take it all. It made her feel very sick and blistered her mouth. Mrs. Connell also noticed that even the bedclothes were burnt where a few drops of the liquid were spilt.

She decided to inform the police, but Wilson had suddenly disappeared. As luck would have it, however, some time afterwards a policeman met and recognized her in the street. She was subsequently tried, but the jury considered the evidence insufficient and she was accordingly discharged. Meanwhile, the C.I.D. had been making inquiries and they found that wherever Wilson had lived there had been mysterious deaths. It transpired that in 1853 she had been living as housekeeper to Captain Peter Mawer, a master-mariner of Boston, who took colchicum for his gout. He died suddenly in 1854 and bequeathed all his property to Catherine Wilson.

Two years later Wilson was living with a man called Dixon in the house of a Mrs. Soames of Bedford Square. Dixon died suddenly in mysterious circumstances, but Wilson remained, being on friendly terms with Mrs. Soames. About Christmas, 1855, Mrs. Soames inherited some money from a half-brother. Not long afterwards she was taken ill with attacks of vomiting and became so weak that a doctor was called in. Wilson nursed her with unswerving devotion and even took charge of the medicine and administered every dose herself. Despite her constant care (or because of it) Mrs. Soames died in agony a few days later. The doctor at first refused to give a death certificate, but, after the autopsy, he certified that death was due to natural causes.

At the trial Dr. Taylor deposed that all Mrs. Soames's symptoms pointed to colchicum having been administered in large and repeated doses. The same symptoms were now recognized as having been present in the case of Mr. Dixon, who had been living with Wilson in Mrs. Soames's house.

Despite the fact that there was no proof that Wilson had either obtained possession of or administered colchicum, she was found guilty and sentenced to death. Soon afterwards it was found that a Mrs. Jackson, with whom Wilson had lived on friendly terms at Boston, had died after four days' illness with similar symptoms and that she had £120 in her possession before her death. Her body was exhumed in 1860, but no trace of colchicum or other poison was detected in the stomach. Nor was this the end, for it was subsequently discovered that a Mrs. Atkinson, a milliner of Kirkby Lonsdale (who always stayed with Wilson whenever she came to London to make business purchases) suddenly died when she was with her in 1860. When her husband arrived in response to a telegram he asked what had become of the £100 in notes which his wife had sewn in her clothes for the purchases, but he was happily reassured by Wilson who explained to him that Mrs. Atkinson had told her that she had been robbed at Rugby on her way to London. Mrs. Atkinson's body was exhumed in May 1852, but again no trace of poison was discovered. Catherine Wilson was thus responsible for five deaths with colchicum. Had she succeeded—as she almost did—with the sixth, it seems unlikely that her career of crime would ever have been revealed. Her fiendish cunning was such that she appeared always in the guise of a ministering angel. It requires but little imagination to see her holding the hand of each of her victims and gazing into their eyes with tender light, whilst they in turn paused in their agony to express their heartfelt gratitude, almost with their last breath, for such devoted nursing, so seemingly devoid of self.

It was in keeping with her character that she neither made any confession nor showed the slightest compunction for her crimes. At least twenty thousand people were present at her execution and she faced them all without a trace of emotion on her face, apparently as indifferent to them as she was at the thought of her cold-blooded and fiendish murders.

### The Bravo Case

I shall now recount for you the strange mystery of the Bravo case. Mr. Bravo, a young barrister, married a wealthy and attractive young widow, a Mrs. Ricardo. It was known that they did not agree very well, particularly as Mrs. Ricardo had informed her husband of a former lover, and this rankled continually in his mind. On Tuesday, April 18th, 1876, after breakfast at their home at Balham, Mr. Bravo drove his wife into town, and on their way a very unpleasant altercation ensued. After arriving in town, Mr. Bravo spent a normal day, had lunch at a restaurant with one of his wife's relatives, and later walked to Victoria Station with a friend and fellow barrister, whom he invited for dinner on the morrow. On arriving home at 4.30 he went for a ride, but the horse bolted and carried him a long distance, so he arrived home tired and exhausted. At 6.30 he was observed to be leaning forward in his chair looking ill. He ordered a hot bath and when getting into it he put his hand to his side and cried aloud with pain. He seemed ill all through dinner, but this does not appear to have been noticed by his wife or her companion, a Mrs. Cox. All partook of much the same food at dinner, but Mr. Bravo was the only one who drank burgundy, the others having sherry and marsala. The butler had decanted the burgundy for some time before dinner was served. The wine was kept in a cellaret in the dining-room and before Mr. Bravo's illness it had been opened once by Mrs. Cox, his wife's companion and once by the maid, and it was opened subsequently by Mrs. Cox who went to fetch some wine for Mrs. Bravo.

At 8 o'clock the party adjourned to the morning room and retired to bed at 9, the ladies to their rooms, Mr. Bravo to his. After the maid had taken wine and hot water to her mistress's bedroom at 9.30 she met her master at the foot of the stairs and thought he looked queer and strange in the face, but he did not appear to be in pain. He usually spoke to her, but on this occasion he merely looked at her twice and passed on. Mr. Bravo, according to Mrs. Cox, then entered his wife's dressing-room and spoke to her in French with reference to the wine. This had frequently been the subject of unpleasant remarks, but when questioned about this particular conversation, Mrs. Bravo said she had no recollection of it.

After this discussion on the wine, Mr. Bravo, Mrs. Bravo and Mrs. Cox all retired to their respective bedrooms. A quarter of an hour later Mr. Bravo opened his door and shouted to his wife: " Florence! Florence! Hot water! " The maid then ran into Mrs. Bravo's room calling out that Mr. Bravo was ill. Mrs. Cox, who had not yet undressed, ran at once into his room and found him standing at the open window and apparently vomiting. She later averred that Mr. Bravo said to her: " I have taken poison. Don't tell Florence." After further severe vomiting Mr. Bravo sank to the floor unconscious and remained so for some hours. A doctor had been sent for, but as soon as Mrs. Bravo arrived on the scene she was very excited and insisted on another doctor being sent for who lived nearby. Both doctors duly arrived and endeavoured to get him to swallow restoratives, but he was unable to do so. Then, as his condition was very critical, they sent for Dr. George Johnson of King's College Hospital. He concluded that some irritant poison had been taken. At about 3 a.m. the patient recovered consciousness and was asked at once: " What have you taken? " But he declared most solemnly and persistently that he had taken nothing except some laudanum for toothache. He remained conscious until he died fifty-five and a half hours after the onset of his illness. His room was then searched, but no trace of poison could be found. There was only the laudanum and chloroform for toothache, and it is noteworthy that Mrs. Cox, the companion, told one of the doctors that she thought he had poisoned himself with chloroform. Autopsy showed all the signs of a severe irritant poison. All the vomit had been thrown away, but, fortunately, some vomit had remained on the leads of the house beneath the bedroom window, though the rain had washed most of it away. That which remained was collected and handed to Professor Redwood, who found a large amount of antimony. This poison was also found in the liver and other parts of the body, and it was concluded that altogether nearly forty grains had been swallowed.

Despite the most careful investigation and inquiries the possession of antimony could not be traced to anyone, nor could it be discovered how the poison had been obtained or administered. The Bravo case will probably remain forever, therefore, among the unsolved poison mysteries. There are, however, some points about which we are given no information and which clearly give food for reflection. We are only told that Mr. Bravo was a sane, healthy young man of thirty who was as normal as any young barrister could be. We are given no information with regard to his wife's previous history. How did her first husband die? Why was his body never exhumed? How long had her companion, Mrs. Cox, been associated with her, and what was her previous history? How did her husband die? Was she very devoted to Mrs. Bravo? Why did she tell the doctors that she felt sure that Mr. Bravo had poisoned himself with chloroform, when in reality he had been poisoned with antimony? Why did she say throughout the whole course of the proceedings that Mr. Bravo had confessed to her that he had poisoned himself, whereas in all the hours that remained of his life he stoutly insisted that he had only taken the laudanum for toothache? If he had wanted to poison himself would he have chosen one of the most painful and distressing irritant poisons when he had only to reach out his hand and drink enough of the toothache drops to sink quietly into the arms of Morpheus for ever? For obvious reasons irritant poisons are rarely taken as a means of suicide by enlightened and cultured people! One question, as always, raises another. What was the butler's reputation? Was he, too, in the service of Mrs. Ricardo before she became Mrs. Bravo? Why did Mrs. Bravo take so long to dress and come on the scene when he flung open the door and cried in agony: " Florence! Florence! Hot water! "? You will say that she was excited and insisted on sending for a practitioner near at hand, but, as the record of poison cases shows, that means nothing!

If I have raised these questions it has not been with the idea of casting suspicion on anyone, but merely to show the logical and persistently thorough way in which the inquiry should proceed in such cases.

(*Part II will appear in the next issue of this* Journal)

## Figures and Tables

**Figure f1:**